# Supplementary material for: Co-translational protein targeting facilitates centrosomal recruitment of PCNT during centrosome maturation in vertebrates
Source: eLife. 2018 Apr 30;7:e34959. doi: 10.7554/eLife.34959 (PMC5976437; doi:10.7554/eLife.34959)
Supplement: Supplementary file 2. — The sense strand of the target site is shown. [file elife-34959-supp2.docx]

| **Target gene** | **Target site (5’ to 3’)**  (PAM: underlined) | **Oligonucleotides for annealing (5’ to 3’)** | |
| --- | --- | --- | --- |
| Zebrafish *pcnt* | CCG**GTCAACAGAAGAGCGCCACC** | TA**GGTGGCGCTCTTCTGTTGAC** | AAAC**GTCAACAGAAGAGCGCCA** |
