# Supplementary material for: Co-translational protein targeting facilitates centrosomal recruitment of PCNT during centrosome maturation in vertebrates
Source: eLife. 2018 Apr 30;7:e34959. doi: 10.7554/eLife.34959 (PMC5976437; doi:10.7554/eLife.34959)
Supplement: Supplementary file 3. [file elife-34959-supp3.docx]

## **Primers for amplifying the zebrafish *pcnt* CRISPR target region**

| **Target region** | **Oligonucleotides for PCR (5’ to 3’)** | | **Amplicon size (bp)** |
| --- | --- | --- | --- |
| *Zebrafish pcnt* | GATGAAGTTGTCGCATGTTTGG | CCTGGGATACAAAAATAGCATACAGACTTTG | 481 |

## **Primers for generating antisense probes for *in situ* hybridization**

| **Probe name** | **Oligonucleotides for RT-PCR (5’ to 3’)** | |
| --- | --- | --- |
| Zebrafish *pcnt-001* | GAAATTAATACGACTCACTATAGGGGGAGGAGTCACAACAGGTAACTAAAGAAC | GAAATCATTAACCCTCACTAAAGGGAATCATGTTGGTGCACTTCACCGA |
| Zebrafish  *pcnt-002* | GAAATTAATACGACTCACTATAGGGGCTGCATAGTGATGTTAAAGAGTTGGAG | GAAATCATTAACCCTCACTAAAGGGAACAGTTTCTCATTAGCGTGTTCC |
| Zebrafish  *cep152* | GAAATTAATACGACTCACTATAGGGGACTCGACACAGAATACAGCAGATGG | GAAATCATTAACCCTCACTAAAGGGAACTGCTTTGTGAGCGTCTCAGTTC |
| Zebrafish  *cep192* | GAAATTAATACGACTCACTATAGGGCCAGTTTTTGCAGGAAGAGCAGATTC | GAAATCATTAACCCTCACTAAAGGGAACCTACAGATCTTTTGGCTGGATTTGC |
| Zebrafish  *cep215* | GAAATTAATACGACTCACTATAGGGAAGATTACGAGAACCAAATCACAGCAC | GAAATCATTAACCCTCACTAAAGGGAAGGTCTTCAGCTCCATGATATTTCTGTAG |
| Human *PCNT 5’ probe* | GAAATTAATACGACTCACTATAGGGTGGGATGTTCACAAAGGAGTGTGAACAAG | GAAATCATTAACCCTCACTAAAGGGAACCAACTTCCACAGAGTCCAGAAGAG |
| Human *PCNT 3’ probe* | GAAATTAATACGACTCACTATAGGGGCTGCCGAAGGTCGATCTCGTAGC | GAAATCATTAACCCTCACTAAAGGGAATCTGCAGTAAAGCCTTCACGTGCTTC |
| **Note** | TAATACGACTCACTATAGGG: T7 promoter | CATTAACCCTCACTAAAGGGAA: T3 promoter |
