# Supplementary material for: Co-translational protein targeting facilitates centrosomal recruitment of PCNT during centrosome maturation in vertebrates
Source: eLife. 2018 Apr 30;7:e34959. doi: 10.7554/eLife.34959 (PMC5976437; doi:10.7554/eLife.34959)
Supplement: Transparent reporting form [file elife-34959-transrepform.docx]

***eLife’s* transparent reporting form**

We encourage authors to provide detailed information *within their submission* to facilitate the interpretation and replication of experiments. Authors can upload supporting documentation to indicate the use of appropriate reporting guidelines for health-related research (see [EQUATOR Network](http://www.equator-network.org/%20)), life science research (see the [BioSharing Information Resource](https://biosharing.org/)), or the [ARRIVE guidelines](http://www.plosbiology.org/article/info:doi/10.1371/journal.pbio.1000412) for reporting work involving animal research. Where applicable, authors should refer to any relevant reporting standards documents in this form.

If you have any questions, please consult our Journal Policies and/or contact us: [editorial@elifesciences.org](mailto:editorial@elifesciences.org).

**Sample-size estimation**

- You should state whether an appropriate sample size was computed when the study was being designed
- You should state the statistical method of sample size computation and any required assumptions
- If no explicit power analysis was used, you should describe how you decided what sample (replicate) size (number) to use

Please outline where this information can be found within the submission (e.g., sections or figure legends), or explain why this information doesn’t apply to your submission:

We have included the sample sizes for the experiments in the figure legends. We did not compute the sample sizes in advance because there were no reliable methods to anticipate the sample numbers needed.

**Replicates**

- You should report how often each experiment was performed
- You should include a definition of biological versus technical replication
- The data obtained should be provided and sufficient information should be provided to indicate the number of independent biological and/or technical replicates
- If you encountered any outliers, you should describe how these were handled
- Criteria for exclusion/inclusion of data should be clearly stated
- High-throughput sequence data should be uploaded before submission, with a private link for reviewers provided (these are available from both GEO and ArrayExpress)

Please outline where this information can be found within the submission (e.g., sections or figure legends), or explain why this information doesn’t apply to your submission:

Our definition of “biological replications”: the entire experimental procedures performed independently on different dates and sometimes by different persons. Our definition of “technical replications”: the replicates of different samples under the same condition in a given experiment (e.g., different coverslips of the cells treated under the same condition). We indicate the number of independent biological and technical replicates in the corresponding figure legends. We include all the data points in our figures.

**Statistical reporting**

- Statistical analysis methods should be described and justified
- Raw data should be presented in figures whenever informative to do so (typically when N per group is less than 10)
- For each experiment, you should identify the statistical tests used, exact values of N, definitions of center, methods of multiple test correction, and dispersion and precision measures (e.g., mean, median, SD, SEM, confidence intervals; and, for the major substantive results, a measure of effect size (e.g., Pearson's r, Cohen's d)
- Report exact p-values wherever possible alongside the summary statistics and 95% confidence intervals. These should be reported for all key questions and not only when the p-value is less than 0.05.

Please outline where this information can be found within the submission (e.g., sections or figure legends), or explain why this information doesn’t apply to your submission:

We have included information about statistical tests and results in the figure legends corresponding to the experiments. The exact values of N for each experiment and the *p* values are included in the figures or figure legends. Definitions for the summary statistics (e.g., mean) and error bars (e.g., standard deviation and confidence intervals) are indicated in the figure legends. Most figures were plotted as dot plots to show all the raw data values.

(For large datasets, or papers with a very large number of statistical tests, you may upload a single table file with tests, Ns, etc., with reference to sections in the manuscript.)

**Group allocation**

- Indicate how samples were allocated into experimental groups (in the case of clinical studies, please specify allocation to treatment method); if randomization was used, please also state if restricted randomization was applied
- Indicate if masking was used during group allocation, data collection and/or data analysis

Please outline where this information can be found within the submission (e.g., sections or figure legends), or explain why this information doesn’t apply to your submission:

The experiments were not randomized. The investigators were not blinded to allocation during experiments and outcome assessment. We indicate this information in “Statistical analysis” under “Materials and Methods” section.

**Additional data files (“source data”)**

- We encourage you to upload relevant additional data files, such as numerical data that are represented as a graph in a figure, or as a summary table
- Where provided, these should be in the most useful format, and they can be uploaded as “Source data” files linked to a main figure or table
- Include model definition files including the full list of parameters used
- Include code used for data analysis (e.g., R, MatLab)
- Avoid stating that data files are “available upon request”

Please indicate the figures or tables for which source data files have been provided:

The source data used to plot graphs in all figures are included as numerical data in the corresponding Excel files: Figure 2B, Figure 3C, Figure 3- figure supplement 2, Figure 4A, Figure 4D, Figure 4- figure supplement 2, Figure 4- figure supplement 3B, Figures 5A-5C, Figure 6, and Figure 6- figure supplement 1.
